# Comparative Physicochemical Screening and Toxicology of Hydroethanol Extracts of the Parts of *Pterocarpus santalinoides* l'Hér. ex DC. (Fabaceae) in Wistar Rats

**DOI:** 10.1155/2022/5953094

**Published:** 2022-02-25

**Authors:** Aimé Cézaire T. Ayéna, Kossivi Dosseh, Kokou Idoh, Amegnona Agbonon, Messanvi Gbeassor

**Affiliations:** ^1^Faculty of Sciences and Techniques of the University of Abomey-Calavi, 01 BP 526, Cotonou, Benin; ^2^Medicinal Plants Research and Training Center (CERFOPLAM), BP 1515 Lomé, University of Lomé, Lome, Togo

## Abstract

**Aims:**

The aim of this study was to compare the phytochemical profile and acute and chronic toxicity of hydroethanolic extracts of three parts of *P. santalinoides*.

**Methods:**

Seven major chemical groups (alkaloids, flavonoids, saponosides, coumarins, tannins, triterpenes, and steroids) were studied. The single dose limit test of 5000 mg/kg body weight was used to evaluate the acute toxicity of each organic extract. Subacute toxicity was evaluated after daily oral doses of 500 and 1000 mg/kg body weight were administered to rats for 28 days.

**Results:**

At a single dose of 5000 mg/kg, none of the extracts (leaf, trunk bark, and root) caused death in experimental rats. However, the trunk bark extract of *P. santalinoides* induced coat change and lethargy in treated rats. Macroscopic observation of the internal organs (liver and kidneys) of the rats showed no abnormalities. In the subacute test, only the trunk extracts induced signs of toxicity such as mobility disorders, diarrhea, and loss of body weight at a dose of 1000 mg/kg.

**Conclusion:**

This study showed that the hydroethanol extracts of the leaves, trunk bark, and root bark of *P. santalinoides* divergently concentrated the main chemical groups of interest. Administration of a single dose of extracts from all three *P. santalinoides* is not toxic to the consumer. However, when used over a long period of time, they can have a harmful effect on the consumer. In view of the different results of the trunk bark extract and in a context of conservation of the species, we recommend the use of the hydroethanolic extract of the leaves in the different treatments in which the three organs are involved.

## 1. Introduction 

Africa is a continent endowed with a rich and very diversified flora that the populations take advantage of to satisfy their basic needs. To cover their food needs, rural African populations resort to subsistence agriculture, which they supplement with edible wild species [1]. In addition to this nutritional function, humans discovered the healing power of plant species [2]. These plants are valuable therapeutic resources that about 80% of the African population uses for primary healthcare [3, 4]. However, although the use of herbal recipes can be considered safe with no major toxicity risks, some are known to be toxic in high doses and others can have potentially harmful effects after prolonged use [5]. It is therefore essential that the prospects for the development of improved traditional medicines be oriented towards the evaluation of the safety of the plant extracts used in the preparation of these medicinal recipes.

*Pterocarpus santalinoides* Her. ex. DC. is a species of the Fabaceae family [6]. Native to Nigeria, the species has spread throughout the West African subregion. It is known by the vernacular names Gbègbètin (fon), Gbèngbèn, Ewè Aègbè, and Tigbi (yoruba, nago), producing fruits for sowing food [6, 7]. It is a plant species of great food and medicinal utility in Africa and Benin. The work of Ayena et al. [2] reports that the leaves, trunk bark, and roots of *P. santalinoides* are in great demand alone or in association with others to treat gastrointestinal disorders (dysentery, diarrhea, vomiting, and abdominal cramps), urinary tract infections, and sexual health conditions. The work of Okoye et al. [8] reports that extracts of *P. santalinoides* leaves have antidiarrheal and antispasmodic effects. The almonds of the fruits of the species, in addition to their food uses, can be used to treat diarrhea and dysentery. Consumed with colas (*Cola nitida*; Sterculiaceae), boiled almonds contribute to the treatment of gastrointestinal ulcers. Consuming them in combination with honey has a beneficial effect on the consumer's memory. The work of Anowi et al. [9] showed that the extract of the leaves of the species was rich in tannins, flavonoids, terpenoids, steroids, alkaloids, glycosides, saponins, and resins which would justify its use as an analgesic product. This study also had the merit of showing that the extract of *P. santalinoides* leaves can be safely consumed. In spite of the strong involvement of the parts of *P. santalinoides* in medicinal preparations, only the toxicological study of the leaves was carried out. It is therefore questionable what the toxicological profile of the other parts (trunk bark and roots) is if they are also used as leaves. The objective of this study is to evaluate the acute and subacute toxicity of the hydroethanolic extract of the 3 parts (leaves, trunk, and root bark) of *P. santalinoides* on rats of Wistar strains.

## 2. Materials and Methods

### 2.1. Plant Material and Extraction

The study material consisted of roots, leaves, and stem bark of *P. santalinoides*. The collection was carried out in the period from April to May 2014 in the commune of Adjarra in southern Benin. The specimen was identified and certified by Professor Hounnankpon Yedomonhan, curator of the National Herbarium of the University of Abomey-Calavi, under the reference number 10185509.

After washing with distilled water, the samples were dried in the laboratory at a temperature of 25 ± 1 and then ground to a fine powder using an electric grinder (Brook Crompton Series 2000, Germany). The extraction was carried out by soaking 50 grams of the powder of each part of the plant in 500 ml of a water-ethanol solvent (80–20: v/v) under continuous stirring for 72 hours. The following formula was used to determine the yield (*R*) of the extraction:

(1) $$\begin{array}{c} R\left(\%\right)=\frac{\mathrm{Me}\;}{\mathrm{Mv}}\;x\;\mathrm{100}, \end{array}$$

where *R* (%) is yield in %; Me is mass of the extract after solvent evaporation; and Mv is mass of plant material used for extraction.

### 2.2. Experimental Animals

Wistar rats weighing between 130 and 145 g (males and females) provided by the Department of Physiology/Pharmacology of “Université de Lomé” were used. All methods and protocols used in this study were observed following the established public health guidelines “Guide for Care and Use of Laboratory Animals” [10, 11].

### 2.3. Ethical Approval

This study is part of a thesis. The committee of the “Doctoral School Life and Earth Science (ED-SVT)” of the University of Abomey-Calavi (UAC, Benin) under the number 10185509 has authorized this study.

### 2.4. Phytochemical Studies of the Three Parts of *P. Santalinoides*

The research of the great chemical groups in the 3 plant extracts was made by a summary qualitative phytochemical analysis from the staining tests [12].

### 2.5. Highlighting of the Alkaloids

#### 2.5.1. Alkaloids

The tests are performed by precipitation reactions with Dragendorff's reagent. The appearance of an orange precipitate will reveal the presence of alkaloids.

### 2.6. Detection of Anthracenosides

In a test tube, the extracts were first subjected to the preparation of an ethereal (yellow) phase and then the potassium hydroxide (KOH) solution was added. The presence of anthraquinones led to the red coloration of the aqueous phase.

### 2.7. Detection of Flavonoids

2 to 3 drops of a diluted iron perchloride (FeCl_3_) solution are added to a few ml of extract solution in a test tube. The observation of a greenish coloration indicates the presence of flavonoids.

### 2.8. Detection of Saponosides

After shaking a test tube containing 2 ml of aqueous extract for one minute, a persistent foam of about 1 cm in height is formed in the presence of the saponosides.

### 2.9. Highlighting of Tannins

1 ml of the 10% aqueous lead acetate solution was added to 3 ml of extract. The formation of a blue, blue-black, whitish, or brownish precipitate indicates the presence of tannins.

### 2.10. Detection of Triterpenes and Steroids

A mixture of methanol and chloroform was added to the nonhydrolyzed vegetable powder, and then a few drops of sulfuric acid were added to the top of the extract suspension. The development of a red-brown or orange ring at the separation of the 2 liquids indicates their presence.

### 2.11. Acute Toxicity Test

Limit test dose of 5000 mg/kg was used as indicated by Organization for Economic Cooperation Development (OECD) guidelines [10]. Three female rats, each sequentially dosed at intervals of 48 h, were used for the test. Animals were observed individually during 4 h for immediate signs of toxicity and mortality and at least once daily for 14 days for delayed toxic symptoms and mortality.

### 2.12. Subacute Toxicity Test

Oral repeat-dose toxicity study was carried out according to OECD guideline 407 [13]. Animals were divided into seven groups of 5 animals each. Group 1 received 10 ml/kg body weight of distilled water and served as control. Groups 2 to 7 received extract at two doses of 500 and 1000 mg/kg body weight, respectively. The test animal groups (2 to 7) were treated at the same time for 28 days by gastric gavage using two doses (500 and 1000 mg/kg). These doses were selected among the active pharmacological doses recorded during the work presented in another thesis study [11]. During the experiment, the animals were weighed every seven days (day 0, day 7, day 14, day 21, and day 28), followed, and observed individually twice a day (in the morning and the afternoon). A data collection sheet was prepared for each rat in order to collect any signs of toxicity (changes in skin, hair, etc., edema, backward walking, breathing difficulties, and morbidity mortality). At the end of treatment, the rats were deprived of the food the last night before sampling. The blood samples on day 29 were taken by puncture at the sinus for all animals under ether anaesthesia.

### 2.13. Hematological and Biochemical Analysis

On the 29th day, after an overnight fast, the rats were anaesthetized with ether, and blood samples for hematological and biochemical analysis were collected into tubes with and without EDTA, respectively. Hemoglobin, hematocrit, red blood cell count, white blood cell count, mean corpuscular hemoglobin concentration (MCHC), mean corpuscular hemoglobin (MCH), mean corpuscular volume (MCV), and platelet count were determined using an automatic counter (Sysmex K21, Tokyo, Japan). Biochemical analysis was performed on serum obtained after centrifugation of total blood (without anticoagulant) at 3000 rpm for 10 min. Standardized diagnostic kits (Labkit®) were used for spectrophotometric determination of the following biochemical parameters: alanine aminotransferase (ALT), aspartate aminotransferase (AST), creatinine, alkaline phosphatase, total bilirubin (TB), total proteins, and urea.

Necroscopy of all animals was carried out and the organ weights (heart, livers, lungs, and kidneys) were recorded.

### 2.14. Statistical Analysis

The results are expressed as mean ± standard error of the mean (SEM). Statistical analysis was performed by one-way analysis of variance (ANOVA) with Tukey's test to evaluate significant differences between groups. *p* values < 0.05 were considered significant. All statistical analyses were carried out using Excel.

## 3. Results

The extracts obtained by maceration of the leaves, bark of the trunk, and roots of *P. santalinoides* were of green and brown color with respective yields of 11.59%, 12.09%, and 9.59%, respectively, relative to the initial powder weight.

### 3.1. Phytochemical Components of the Parts of *P. santalinoides*

The results obtained reveal the presence of the compounds sought (alkaloids, flavonoids, saponosides, coumarins, tannins (gallic, catechic), triterpenes, and steroids). The catechic tannins are in the form of traces in all the parts like the coumarins in the trunk and root bark. There is an absence of anthraquinones (Table 1).

### 3.2. Acute Toxicity

#### 3.2.1. Assessment of Behavioural Parameters

Oral administration of a single dose of 5000 mg/kg of the three extracts (leaf, trunk bark, and root bar) of *P. santalinoides* did not induce any signs of acute toxicity was not observed for a short period (48 h) and throughout the experimental period. No diarrhea, drowsiness, reduced mobility, weight loss or decreased sensitivity.

#### 3.2.2. Determination of LD50

After 14 days of observation, no deaths were observed in treated rats, which leads to the conclusion that the Median Lethal Dose (LD_50_) is greater than 5000 mg/Kg body weight (Table 2).

### 3.3. Subacute Toxicity

Daily oral administration of hydroethanolic extracts from the parts of *P. santalinoides* for 28 days does not cause any lethality or disturbance in rats, except for the batch treated with trunk extracts. Rats treated with trunk extracts at 1000 mg/kg showed signs of toxicity such as impaired mobility, diarrhea, and body weight loss. These signs appeared from the 7th day of treatment and remained constant until the 28th day.

### 3.4. Influence of the Hydroethanolic Extract of the Parts of *P. santalinoides* on the Weight Evolution

**Figure 1:**
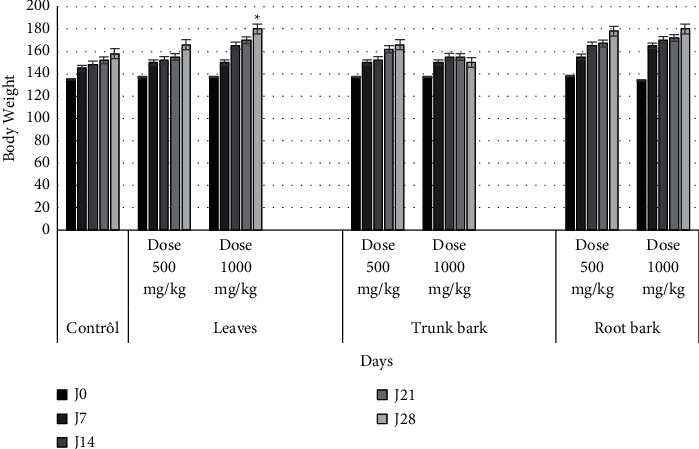
Effects of the hydroethanolic extract of the parts of *P. santalinoides* leaves on the evolution of rat weight.

The rats used in this study were randomized into several batches of homogeneous weight (133 ± 3.86 g, 135.9 ± 5.64 g, and 136.1 ± 5.04 g) for leaf, trunk, and root extract batches, respectively. The weight of the control batches is 135 ± 2.34 g. The weight of the experimental animals varied during the experiment until the end of the 4th week. The mean weight of the control lots increased from 135 ± 2.34 g to 169.1 ± 4.15 g, an increase of 34 g. Those of the lots treated with 500 and 1000 mg/kg extract were 170.0 ± 5.39 g and 183.1 ± 4.01 g for leaf extract (Figure 1); 153.0 ± 2.80 g and 139.1 ± 3.6 g for trunk bark (Figure 1); and 168.1 ± 4.2 g and 166.3 ± 2.4 g for root bark (Figure 1). Overall, these values indicate that body weight gain is recorded in animals treated with leaf and root bark extracts. A significant difference (*p* < 0.05) was recorded in the body weight of rats treated with leaf extracts at 1000 mg/kg body weight. On the other hand, data from rats treated with extracts from the bark of the trunk at 1000 mg/kg indicated a weight loss, although it was not significant (Figure 1).

On the graphs above, each point represents the mean ± standard errors of the mean (SEM), *n* = 5. A statistical difference is observed between the control group and the groups treated with plant extracts at 1000 mg/kg (^*∗*^*p* < 0.05; ANOVA).

### 3.5. Influence of Hydroethanolic Extract of *P. santalinoides* Parts on Relative Weight

After 28 days of treatment with hydroethanolic extract of *P. santalinoides* parts, macroscopic examination of the vital parts of treated rats revealed no change in color or texture. Apart from the relative liver weights of the treated rats, which varied significantly and were dose-dependent, no changes in organ weights were recorded.

In rats treated with leaf extracts and root bark, only the relative liver weight of the 1000 mg/kg body weight batch varied significantly (*p* < 0.05) for leaves (Table 3) and highly significantly (*p* < 0.01) for roots compared to controls (Table 3). In rats treated with trunk extracts, both doses (500 and 1000 mg/kg) caused a highly significant increase in liver weights in treated rats (Table 3).

### 3.6. Effects of the Hydroethanolic Extract of *P. santalinoides* Parts on the Hematological Parameters of Rats

The results of the analyses in relation to the hematological parameters of rats treated with leaf extracts for 28 days show no significant change in hematological parameters compared to the control lot (*p* > 0.05). However, rats treated with trunk bark extract showed a twofold change in mean corpuscular volume (MV) and mean corpuscular hemoglobin concentration (MCHC). The GMV of treated rats decreased significantly (*p* < 0.05) at the 500 mg/kg dose and highly significantly (*p* < 0.0001) at the 1000 mg/kg body weight dose. The CCMH, on the other hand, increased in a highly significant manner (*p* < 0.001) at both doses tested. The hydroethanolic extract of *P. santalinoides* root bark did not induce any significant variation in hematological parameters at the 500 mg/kg dose. On the other hand, the 1000 mg/kg body weight dose induced a statistically significant decrease in hemoglobin level and mean corpuscular hemoglobin concentration (*p* < 0.05; *p* < 0.01) (Table 4).

### 3.7. Effect of Hydroethanolic Extract of *P. santalinoides* Parts on Biochemical Parameters in Rats

Daily oral administration of hydroethanolic extracts from the leaves and trunk bark of *P. santalinoides* at doses of 500 and 1000 mg/kg for 28 days did not cause any significant change (*p* > 0.05) in blood enzyme (AST, ALT, and PAL), creatinine, total bilirubin, and total protein levels in rats in the treated groups compared to those in the control group (Table 3). On the other hand, at the urea level, the results indicate a very highly significant (*p* < 0.001) decrease in values in rats treated at both doses (500 and 1000 mg/kg) compared to the control group (Table 5).

As for the root bark extract, in addition to the very highly significant decrease in urea levels at the 1000 mg/kg dose (^∗∗∗^*p* < 0.001), the extract caused a significant (^*∗*^*p* < 0.05) increase in total protein concentration in treated rats (Table 5).

## 4. Discussion

In the case of acute toxicity, the work of Diezi [13] had shown that substances with an LD_50_ greater than 5000 mg/kg are practically nontoxic. Based on this classification, it follows that hydroethanolic extracts from the parts (leaves, trunk, and root bark) of *P. santalinoides* do not show oral toxicity. The results of Anowi et al. [9] had shown that the leaves of the species did not cause any signs of toxicity at 5000 mg/kg. They deduced, as in the present study, that the LD_50_ dose is higher than 5000 mg/kg. According to the toxicity scale of Hodge and Sterner [14], for an LD_50_ between 5000 and 15000 mg/kg, the corresponding dry matter mass for a 12.5 kg child is 180 g, which is equivalent to a consumption of 1.008 kg of dry matter of *P. santalinoides* for a 70 kg adult. These data indicate that the hydroethanolic extract of the parts of *P. santalinoides* is safe to use.

The results of the subacute toxicity study show that there is a variation in the body weights of the treated animals compared to the controls whatever the dose administered. The data obtained indicate an increase in the weight of rats treated with leaf and root bark extracts. Although this was only significant (*p* < 0.05) for the leaf extract at 1000 mg/kg body weight, this increase in weight could be related to a stimulation of the animals' appetite by the extract, which would result in an increase in their food consumption [15]. The same result was obtained by Pieme et al. [16] on rats treated for 26 days with the aqueous extract of *Senna alata*.

As for the rats treated with trunk extracts, a weight loss of the rats coupled with diarrhea was recorded. This state can be explained by the irritating effect exerted on the intestinal tract by the saponosides contained in the hydroethanolic extract of the trunk of the species [17]. Indeed, diarrhea causes a loss of water, electrolytes, and a decrease in the absorption of nutrients. Also, tannins are phenolic compounds with the power to form indigestible complexes with nutrients in food or with proteins in the body such as digestive enzymes [17]. Their presence in this extract can, by interacting with the food consumed, induce a decrease in the weight of treated rats. The result is an inhibition of the absorption of complexed nutrients as well as a decrease in the activity of digestive enzymes and therefore a reduction in nutritional performance.

According to the FAO [18], the action of these natural compounds may continue for 15 days after the last ingestion of the product containing them.

The organ data recorded in relation to a decrease in relative liver weight in rats treated with leaf and root extracts and a gain in relative liver weight in rats treated with trunk extract are consistent with body weight gain of the former and weight loss of the latter. In addition, the change in relative liver weight associated with the presence of alkaloids may induce hepatotoxic activity [19].

With regard to hematological parameters, the results obtained indicate that long-term consumption of this extract induces a decrease in GMV, which will lead to a normochromic microcytic anemia. As for the root extracts, the highly significant decrease in MCCH is associated with the hemoglobin level which also decreased at the 1000 mg/kg body weight dose. These joint results lead to the conclusion that long-term consumption of this extract could induce anemia.

The liver ensures the synthesis of urea from ammonia, which comes from the catabolism of proteins [20]. A decrease in the concentration of urea in the blood is often associated with cirrhosis of the liver or acidosis. The results of this study show that the biochemical parameters measured (AST, ALT, creatinine, total bilirubin, and total protein) did not vary significantly for root extracts at 500 mg/kg body weight. However, at 1000 mg/kg body weight, the extracts from the three parts (leaves, trunk, and root) caused a significant decrease in the concentration of urea and total protein. Moreover, the decrease in urea concentration was not associated with an increase in the concentration of liver enzymes (ASAT, ALAT, and PAL) in the blood, thus excluding the case of cirrhosis of the liver. The results thus obtained indicate that, at a dose of 1000 mg/kg, all the extracts taken together are likely to induce acidosis during use.

## 5. Conclusion

This comparative study of the phytochemical and toxicological potential of hydroethanolic extracts (80%) of the organs (leaves, trunk, and root bark) of *P. santalinoides* revealed that they variously concentrated 7 major chemical groups. None of the extracts showed toxicity at a single dose of 5000 mg/kg by the oral route. However, at repeated doses (500 mg/kg and 1000 mg/kg), the hydroethanol extract of the parts (trunk and root bark) of *P. santalinoides* induced anemia and acidosis. Following this work, only the extract of the leaves can be consumed without risk for the populations. As for the two other extracts, histological studies are necessary to bring more information on the hepatotoxicity of the extracts of this biological resource.

## Figures and Tables

**Table 1:** Phytochemical components of *P. santalinoides*.

Major chemical groups		Leaf extract	Bark trunk extract	Bark root extract
Alkaloids		+	+	+
Flavonoids		++	+++	±
Saponosides		+++	+++	+++
Coumarines		+	±	±
Tannins	Gallic	++	+++	+
	Catechic	±	±	±
Triterpenes and steroids		+++	++	+++

*Note.* ± = trace; + = presence; ++ = important; +++ = very important.

**Table 2:** Mean body weight of rats after 28-day treatment with hydroethanolic leaf, bark of trunk, and of bark root of *P. santalinoides*.

Week	Mean body weight (g ± SEM)						
	Control	Leaves extract		Bark of trunk extract		Bark of root extract	
	(Normal saline)	500 mg/kg	1000 mg/kg	500 mg/kg	1000 mg/kg	500 mg/kg	1000 mg/kg
0	135,3 ± 2,34	133,2 ± 3,86	135,4 ± 3,86	134,9 ± 5,64	133,9 ± 5,40	135,1 ± 4,01	136,1 ± 3,04
1	143,6 ± 3,20	144,5 ± 3,30	146,7 ± 2,80	140,3 ± 4,60	139,7 ± 4,20	143,3 ± 2,50	140,0 ± 2,70
2	147,2 ± 1,40	152,1 ± 2,50	160,9 ± 3,40	147,1 ± 3,10	143,4 ± 3,90	154,7 ± 4.10	150,2 ± 2,10
3	160,5 ± 4,20	164,3 ± 4,30	175,4 ± 3,70	154,2 ± 2,40	147,2 ± 3,10	158,3 ± 3,60	156,1 ± 3,20
4	169,1 ± 4,15	170,0 ± 5,39	183,1 ± 4,01	160,0 ± 2,80	139,1 ± 3,60	168,1 ± 4,20	166,3 ± 2,40

Values are expressed as mean ± SEM (*n* = 5); *p* > 0.05; control group versus extract.

**Table 3:** Mean organ weight of rats after 28-day treatment with hydroethanolic leaf, bark of trunk, and bark of root of *P. santalinoides*.

Parts	Mean relative weight of the parts (g ± SEM)						
	Control	Leaves extract		Bark of trunk extract		Bark of root extract	
	(Normal saline)	500 mg/kg	1000 mg/kg	500 mg/kg	1000 mg/kg	500 mg/kg	1000 mg/kg
Heart	0,340 ± 0,01	0,352 ± 0,01	0,296 ± 0,02	0,376 ± 0,02	0,338 ± 0,01	0,36 ± 0,01	0,33 ± 0,01
Liver	3,068 ± 0,23	3,008 ± 0,12	2,606 ± 0,2^*∗*^	3,728 ± 0,37^∗∗∗^	3,776 ± 0,12^∗∗∗^	2,82 ± 0,11	2,56 ± 0,08^∗∗^
Kidney	0,298 ± 0,00	0,334 ± 0,01	0,278 ± 0,01	0,390 ± 0,04	0,470 ± 0,05	0,29 ± 0,00	0,31 ± 0,01
Lung	0,650 ± 0,03	0,732 ± 0,06	0,582 ± 0,04	0,848 ± 0,1	0,883 ± 0,05	0,58 ± 0,02	0,64 ± 0,02

Values are expressed as mean ± SEM (*n* = 5); ^*∗*^*p* < 0.05, ^∗∗^*p* < 0.01, and ^∗∗∗^*p* < 0.001; control group versus extract.

**Table 4:** Hematological parameters for rats after 28-day treatment with hydroethanolic leaf, trunk of bark, and root of bark of *P. santalinoides*.

Parameters	Control	Leaves extract		Bark of trunk extract		Bark of root extract	
	(Normal saline)	500 mg/kg	1000 mg/kg	500 mg/kg	1000 mg/kg	500 mg/kg	1000 mg/kg
WBC (10^3/^µL)	7,42 ± 0,38	7,26 ± 1,11	8,64 ± 2,14	8,04 ± 1,01	9,24 ± 0,44	6,90 ± 0,59	5,86 ± 0,65
RBC (10^6^/µL)	8,178 ± 0,23	8,064 ± 0,15	8,124 ± 0,22	8,782 ± 0,18	8,668 ± 0,10	8,10 ± 0,10	7,47 ± 0,14
HGB (g/dL)	16 ± 0,33	15,78 ± 0,19	15,96 ± 0,36	17,10 ± 0,19	16,22 ± 0,22	16,16 ± 0,16	14,56 ± 0,43^*∗*^
HCT (%)	48,1 ± 0,78	48,72 ± 0,45	49,00 ± 1,26	47,30 ± 0,52	45,22 ± 0,51	49,80 ± 0,73	45,94 ± 1,47
MCV (fL)	58,84 ± 1,03	60,52 ± 0,89	60,44 ± 0,84	54,00 ± 1,10^*∗*^	52,28 ± 0,68^∗∗∗^	61,36 ± 0,83	60,66 ± 0,88
MCH (pg)	19,5 ± 0,41	19,54 ± 0,19	19,6 ± 0,25	19,42 ± 0,32	18,66 ± 0,13	19,86 ± 0,16	19,14 ± 0,18
MCHC (%)	33,22 ± 0,27	32,36 ± 0,36	32,52 ± 0,19	36,00 ± 0,15^∗∗∗^	35,8 ± 0,33^∗∗∗^	32,40 ± 0,22	31,66 ± 0,25^∗∗^
Platelet (10^3^/*μ*L)	511,60 ± 22,50	643,00 ± 29,23	597,00 ± 49,35	462,00 ± 27,32	450,20 ± 51,56	465,00 ± 20,52	427,40 ± 28,25

Values are expressed as mean ± standard error of mean (SEM) (*n* = 5); ^*∗*^*p* < 0.05, ^∗∗^*p* < 0.01, and ^∗∗∗^*p* < 0.001; control group versus extract. WBC: white blood cells, RBC: red blood cells, HGB: hemoglobin, HCT: hematocrit, MCV: mean corpuscular volume, MCH: mean corpuscular hemoglobin, and MCHC: mean corpuscular hemoglobin concentration.

**Table 5:** Biochemical parameters for rats after 28-day treatment with hydroethanolic leaf, bark of trunk, and bark of root of *P. santalinoides*.

Parameters	Control	Leaves extract		Bark of trunk extract		Bark of root extract	
	(Normal saline)	500 mg/kg	1000 mg/kg	500 mg/kg	1000 mg/kg	500 mg/kg	1000 mg/kg
AST (UI/L)	136,60 ± 23,71	144,60 ± 7,32	146,6 ± 10,23	179,40 ± 30,55	170,2 ± 15,04	125,40 ± 13,97	130,60 ± 5,44
ALT (UI/L)	42,80 ± 6,94	27,00 ± 2,7	31,20 ± 1,96	61,20 ± 6,28	43,60 ± 5,46	40,60 ± 8,94	36,40 ± 2,78
ALP (UI/L)	220,80 ± 24,73	158,80 ± 12,08	234,00 ± 22,78	179,40 ± 30,55	170,20 ± 15,04	92,00 ± 7,39	51,40 ± 5,62
Creatinine (mg/L)	8,80 ± 0,37	7,80 ± 0,73	8,00 ± 0,31	9,60 ± 0,50	8,20 ± 0,58	9,80 ± 0,37	9,00 ± 0,44
Total bilirubin (mg/dL)	10,20 ± 0,86	10,60 ± 1,2	10,20 ± 0,48	12,60 ± 1,07	16,20 ± 2,78	9,00 ± 0,63	8,80 ± 0,37
Total protein (g/L)	75,40 ± 3,61	73,00 ± 3,2	74,00 ± 1,26	80,00 ± 2,32	76,40 ± 1,96	80,20 ± 2,39	87,40 ± 1,47^*∗*^
Urea (mg/L)	54,80 ± 2,51	35,20 ± 2,51^∗∗∗^	31,20 ± 2,41^∗∗∗^	41,80 ± 2,87^∗∗^	33,80 ± 1,39^∗∗∗^	48,80 ± 2,35	37,60 ± 2,37^∗∗∗^

Values are expressed as mean ± standard error of mean (SEM) (*n* = 5); ^*∗*^*p* < 0.05, ^∗∗^*p* < 0.01, and ^∗∗∗^*p* < 0.001; control group versus extract. AST: aspartate transferase, ALT: alanine aminotransferase, and ALP: alkaline phosphatase.

## Data Availability

All technical and scientific data are incorporated in this manuscript. Nevertheless, the authors are willing to contribute to researchers who would like to reproduce their different tests.
